# A pilot feasibility randomised controlled trial of bone antiresorptive agents on bone turnover markers in critically ill women

**DOI:** 10.1038/s41598-024-52607-1

**Published:** 2024-01-24

**Authors:** Neil R. Orford, Allison Bone, Mark A. Kotowicz, Michael Bailey, Julie A. Pasco, Matthew Maiden, Nima Kakho, Claire Cattigan, Martina Nichonghaile, Claire Jones, Carol Hodgson, Priya Nair, Jacqueline Center, Rinaldo Bellomo

**Affiliations:** 1 Intensive Care Unit, University Hospital Geelong, Barwon Health, Geelong, Australia; 2 Australian and New Zealand Intensive Care Research Centre (ANZIC-RC), School of Public Health and Preventive Medicine (SPPHPM), Monash University, Melbourne, Australia; 3 IMPACT-Institute for Mental and Physical Health and Clinical Translation, School of Medicine, Deakin University, Geelong, Australia; 4 Department of Critical Care, University of Melbourne, Melbourne, Australia; 5 Department of Medicine-Western Health, The University of Melbourne, Melbourne, Australia; 6 Intensive Care Unit, St Vincent’s Hospital Sydney, Sydney, Australia; 7 Garvan Institute of Medical Research, Sydney, Australia

**Keywords:** Biomarkers, Endocrinology, Health care

## Abstract

Critical illness is associated with increased bone turnover, loss of bone density, and increased risk of fragility fractures. The impact of bone antiresorptive agents in this population is not established. This trial examined the efficacy, feasibility, and safety of antiresorptive agents administered to critically ill women aged fifty years or greater. Women aged 50 years or greater admitted to an intensive care unit for at least 24 h were randomised to receive an antiresorptive agent (zoledronic acid or denosumab) or placebo, during critical illness and six months later (denosumab only). Bone turnover markers and bone mineral density (BMD) were monitored for 1 year. We studied 18 patients over 35 months before stopping the study due to the COVID-19 pandemic. Antiresorptive medications decreased the bone turnover marker type 1 cross-linked c-telopeptide (CTX) from day 0 to 28 by 43% (± 40%), compared to an increase of 26% (± 55%) observed with placebo (absolute difference − 69%, 95% CI − 127% to − 11%), p = 0.03). Mixed linear modelling revealed differences in the month after trial drug administration between the groups in serum CTX, alkaline phosphatase, parathyroid hormone, and phosphate. Change in BMD between antiresorptive and placebo groups was not statistically analysed due to small numbers. No serious adverse events were recorded. In critically ill women aged 50-years and over, antiresorptive agents suppressed bone resorption markers without serious adverse events. However, recruitment was slow. Further phase 2 trials examining the efficacy of these agents are warranted and should address barriers to enrolment.

Trial registration: ACTRN12617000545369, registered 18th April 2017.

## Introduction

Osteoporosis is a chronic progressive disease and major public health issue^1^, characterised by low bone mass, micro-architectural bone disruption, and skeletal fragility leading to fracture^2^. The lifetime risk of osteoporotic spine, hip, or wrist fracture is 30–40% in developed countries, and the lifetime risk of hip fracture is one in six in white females^3^, accompanied by a burden of mortality, morbidity, and cost^4,5^. However, as few as 13–27% of patients with osteoporosis are treated following a fragility fracture, suggesting osteoporosis remains an underdiagnosed disease^6^.

Critical illness, with its associated immobilisation, inflammation, and endocrine dysfunction, is associated with an increased risk of osteoporosis due to increased bone turnover, loss of bone mineral density (BMD), and an increased risk of fragility fracture^7–15^. An increased risk of osteoporosis following critical illness would contribute to long-term morbidity and mortality and is a potential target for intervention. The antiresorptive agents zoledronic acid and denosumab are effective at preventing bone resorption in non-critically ill populations through inhibition of osteoclast maturation and activity but have not been systematically assessed in critically ill populations^16^.

Accordingly, we aimed to provide pilot data regarding the efficacy, safety, and feasibility of subcutaneous denosumab or intravenous zoledronic acid in adult women aged 50 years and over with an intensive care (ICU) admission greater than 24-h duration.

## Materials and methods

### Design, ethics and consent

This trial was a prospective, randomised, controlled, clinical trial performed in a single-centre tertiary regional Intensive Care Unit (ICU) in Geelong, Australia. Prior to commencement, approval was obtained from the Barwon Health Human Research Ethics Committee. This study was performed in accordance with the ethical principles of the Declaration of Helsinki, ICH GCP Notes for Guidance on Good Clinical Practice, and Australian National Health and Medical Research Council National Statement on Ethical Conduct in Research Involving Humans 2007 (Updated 2018). Written informed consent was obtained from patients or their medical treatment decision maker prior to enrolment in the study. Patients underwent screening and randomisation between 15^th^ March 2018 and 16^th^ February 2021. Enrolment ceased in February 2021 due to ongoing interruption of research and bone mineral density services relating to the COVID-19 pandemic. The full trial protocol is available in the supplement materials.

### Study population and controls

Eligible patients were women admitted to intensive care and (1) aged 50 years of age or greater, *or* postmenopausal (amenorrhea for greater than 6-months or serum follicular stimulating hormone (FSH) > 40mIU/L) *or* aged less than 50 years or age and had undergone bilateral salpingo-oopherectomy; *and* (2) with an intensive care unit length of stay of 24 h or greater.

Exclusion criteria included (1) active malignancy, (2) metabolic bone disease, (3) pregnancy, (4) expected glomerular filtration rate (eGFR) < 30 ml/min at time of enrolment, (5) contraindication to trial medication, (6) poor oral hygiene, (7) malabsorption syndromes or extensive small bowel resection, (8) current treatment with an anti-resorptive agent, or (9) a current indication for anti-resorptive therapy. The full details of inclusion and exclusion criteria appear in Supplement Table 1.

### Randomisation

Randomisation was performed using a computer-generated allocation schedule developed and adhered to by the site clinical trials pharmacist. All personnel, apart from the trial pharmacist, were blinded to treatment allocation. Prior to July 2019, enrolled patients were randomised 1:1 to either denosumab 60 mg subcutaneous 6-monthly, or placebo. In August 2019, the management committee agreed a third zoledronic acid arm should be added to the pilot trial to investigate the safety and efficacy of this first-line agent in the critical illness setting. Subsequently enrolled patients were randomised 1:1:1 to either denosumab 60 mg subcutaneous 6-monthly, or zoledronic acid 5 mg intravenous injection annually, or placebo.

### Interventions

Prior to administration of trial medication, participant serum vitamin D level was measured. If the level was less than 50 nmol/L, 50,000 IU oral or enteral cholecalciferol cholecalciferol was administered. Serum vitamin D level was reassessed, and repeat dosing of cholecalciferol administered until participant serum vitamin D level exceeded 50 nmol/L. Participants were assessed as eligible to receive trial medication when they were (1) vitamin D replete, (2) assessed as having no new or untreated sepsis, (3) serum ionised calcium greater than 0.9 mmol/L, and (4) eGFR greater than 30 mL/min and not receiving renal replacement therapy.

On the day of trial medication administration, participants received denosumab 60 mg in a single use 1 ml syringe administered as subcutaneous injection, or zoledronic acid 5 mg in 100 ml 0.9% saline administered as an intravenous infusion, or placebo consisting of 0.9% saline subcutaneous and/or intravenous delivered in an identical preparation and method as the trial medication. The trial medication was administered by an ICU bedside nurse or trial nurse who were blinded to the treatment allocation. Monitoring for hypocalcaemia (defined as ionized calcium < 0.9 mmol/L) occurred at 8–12 h and 24 h post trial drug administration. If present, hypocalcemia was treated with parenteral calcium using existing hospital protocols to maintain a target ionized calcium range of 0.9–1.1 mmol/L).

If the participant was discharged to the ward on the day of trial medication administration, monitoring occurred on the first and second day after trial medication administration. At six-months participants were unblinded, and those receiving denosumab returned to hospital and received denosumab as an outpatient.

### Data collection

Data collected included demographics, osteoporosis risk factors, comorbidity, severity of critical illness score, interventions, ICU and hospital length of stay, survival, serum biochemistry, serum bone formation marker: type 1 N-terminal procollagen (P1NP), serum bone resorption marker: collagen type 1 cross-linked c-telopeptide (CTX), and BMD. Data were collected at ICU baseline (demographic data, clinical information, BTMs), day 7 (biochemistry), post-ICU discharge (BMD), 28-days (BTMs, biochemistry), 6-months (BTMs, biochemistry), and 1-year post-ICU discharge (repeat BMD, BTMs, biochemistry). BMD was presented as an absolute value (g/cm^2^), annualised percentage (difference between BMDs divided by initial BMD calculated as an annualised rate). At completion of the study, participants were provided a summary of their osteoporosis risk factors, treatment received, BMD results, and advice regarding ongoing anti-resorptive therapy and monitoring was provided to the participant and their General Practitioner.

### Outcomes

The primary outcome was change in serum CTX from baseline to day 28 after administration of the first dose of trial medication.

Secondary outcomes included longitudinal change in serum CTX, serum P1NP, other biochemical markers, and annualised change in BMD (lumbar spine and dual femoral neck) for the year after ICU discharge.

The primary feasibility outcome was enrolment and retention over time. Safety outcomes included the incidence of (1) severe hypocalcaemia, (2) osteonecrosis of the jaw, (3) new infection (skin, abdominal, urinary tract, respiratory, bacteraemia, sepsis or septic shock), or (4) new renal failure (acute kidney injury was assessed with the use of a five‐category scoring system to evaluate risk, injury, failure, loss, and end‐stage kidney injury (RIFLE))^17^.

### Statistical analysis

Sample size was based on convenience, with 10 participants per treatment arm. All data was assessed for normality. Continuously normally distributed data were reported as mean (± standard deviation), whereas non-parametric data were reported using median (interquartile range [IQR]) or frequency distribution. The primary outcome (change in CTX to day 28) was compared between groups using a student t-test and reported as a percentage change with a 95% confidence interval. Longitudinal data were analysed using mixed linear modelling fitting main effects for treatment and time with an interaction term between treatment and time reflecting change in treatment over the duration of the study. All analyses were performed using SAS version 9.4 (SAS Institute, Inc., Cary, NC, USA) and a two-sided p-value of 0.05 was used to indicate statistical significance. All data generated or analysed during this study are included in this published article [and its supplementary information files].

## Results

### Participants

**Figure 1:**
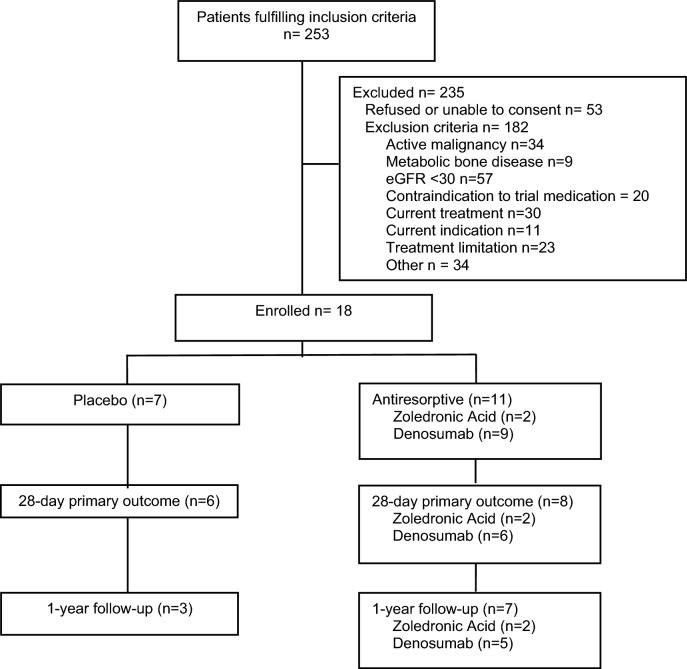
Summary of eligibility, enrolment, and long-term follow-up for study procedures.

We screened 253 patients and enrolled 18 participants over 35 months (9 were randomised to denosumab, 7 to placebo, and 2 to zoledronic acid). Of the 18 participants, 10 (56%) completed 1-year follow-up. Details of screening and flow through the study are provided in Fig. 1. Baseline patient demographics including osteoporosis risk factors, critical illness severity, key interventions and major outcomes are presented in Table 1. Primary osteoporotic risk factors were uncommon.

**Table 1:** Demographic, clinical characteristics, baseline osteoporosis risk factors, biochemistry, interventions, and outcomes by study completion status.

Variable	All
Number	18
Age (yrs)	68.8 ± 7.5
BMI (kg/m^2^)	29.6 ± 6.1
Osteoporosis risk factors	
Parent hip fracture	1 (6)
Previous fragility fracture	0 (0)
BMI < 20 (kg/m^2^)	0 (0)
Rheumatoid arthritis	0 (0)
Alcohol > 3 units/day	0 (0)
Smoker	2 (11)
Secondary osteoporosis	18 (100)
Glucocorticoids	0 (0)
Anti-fracture agents	0 (0)
Chronic disease	
None	16 (89)
Respiratory	1 (6)
Cardiovascular	0 (0)
Liver	0 (0)
Immunosuppressed	
Apache III score	57.8 ± 25.2
ICU admission category	
Surgical	4 (22)
Medical	7 (39)
Cardiothoracic	7 (39)
Interventions	
Mechanical ventilation	15 (83)
Ventilation duration (days)	3.9 [1.7, 8.6]
Glucocorticoid	5 (28)
CRRT	2 (11)
Outcomes	
ICU LOS (days)	8.5 [60, 15.5]
Hospital LOS (days)	20.6 ± 11.6
ICU survival	18 (100)
Hospital survival	18 (100)

Data are shown as mean (± standard deviation), median [interquartile range] or number (%).

*BMI* body mass index, *APACHE* acute physiology and chronic health evaluation; *CRRT* continuous renal replacement therapy, *LOS* length of stay.

### Outcomes

**Figure 2:**
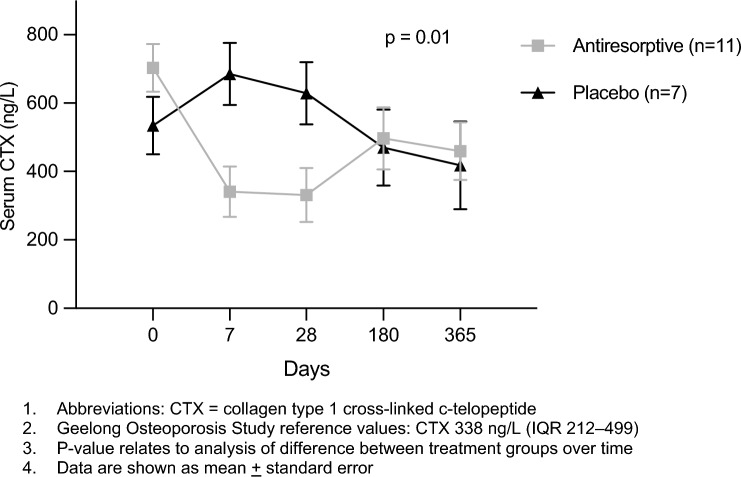
Bone resorption markers in year after critical illness.

The mean serum CTX at day 0 and day 28, absolute and percent change during this period, for the antiresorptive and placebo groups are provided in Table 2. Baseline mean CTX (antiresorptive 666 (± 263) ng/L, placebo 529 (± 221) ng/L) exceeded the third interquartile reference value for population-based women (338 ng/L [IQR, 212–499]^18^. A significant difference in change in serum CTX was observed between the two groups, with a 43% (± 40%) decrease in the antiresorptive group compared to a 26% (± 55%) increase in the placebo group, (difference (95% CI) − 69% (− 127% to − 11%) p = 0.03). This was supported by longitudinal mixed linear modelling of serum CTX over the one-year follow-up period, characterised by a decrease in serum CTX at day 7 and 28 in the antiresorptive group that was no longer apparent at 6 and 12-month follow-up (Fig. 2).

**Table 2:** Change in bone resorption markers over 28-days.

Serum CTX (ng/L*)	Antiresorptive (n = 8)	Placebo (n = 6)	Difference	P-value
Baseline	666 (± 263)	529 (± 221)	–	
Day 28	340 (± 219)	629 (± 243)	–	
Total change	− 326 (± 362)	+ 100 (± 217)	– 426 (− 799 to − 53)	0.03
Percent change	− 43% (± 40%)	+ 26% (± 55%)	– 69% (− 127% to − 11%)	

Data are shown as mean (± standard deviation).

*CTX* collagen type 1 cross-linked c-telopeptide.

**Figure 3:**
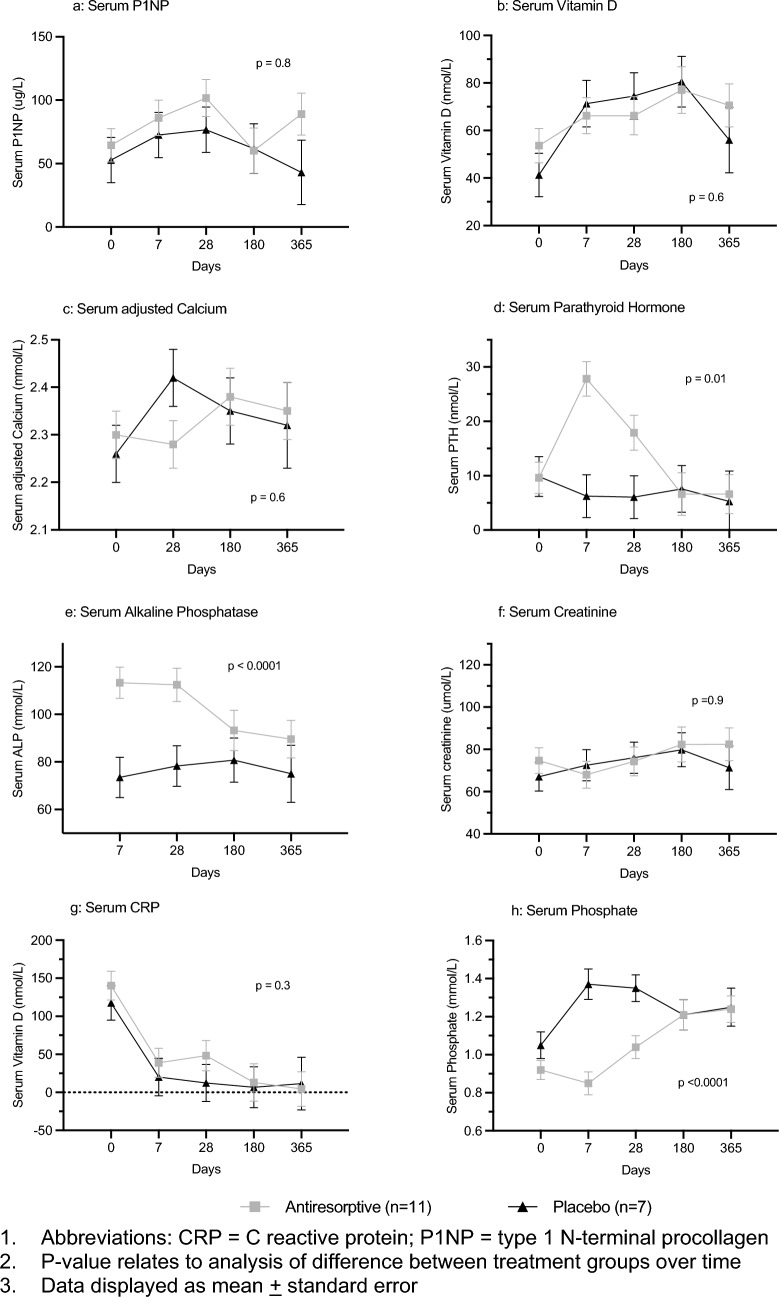
Biochemical, and inflammatory markers over 1 year.

The comparison of distribution biochemical and haematological markers over time for the antiresorptive vs placebo groups are displayed in Fig. 3. There was no difference in the distribution of the bone formation marker (P1NP), vitamin D, serum calcium, serum creatinine, or serum C-reactive protein. However, there was a significant increase in serum parathyroid hormone and alkaline phosphatase and a significant decrease in serum phosphate at the day 7 and 28 time points in the antiresorptive group compared to the placebo group.

**Figure 4:**
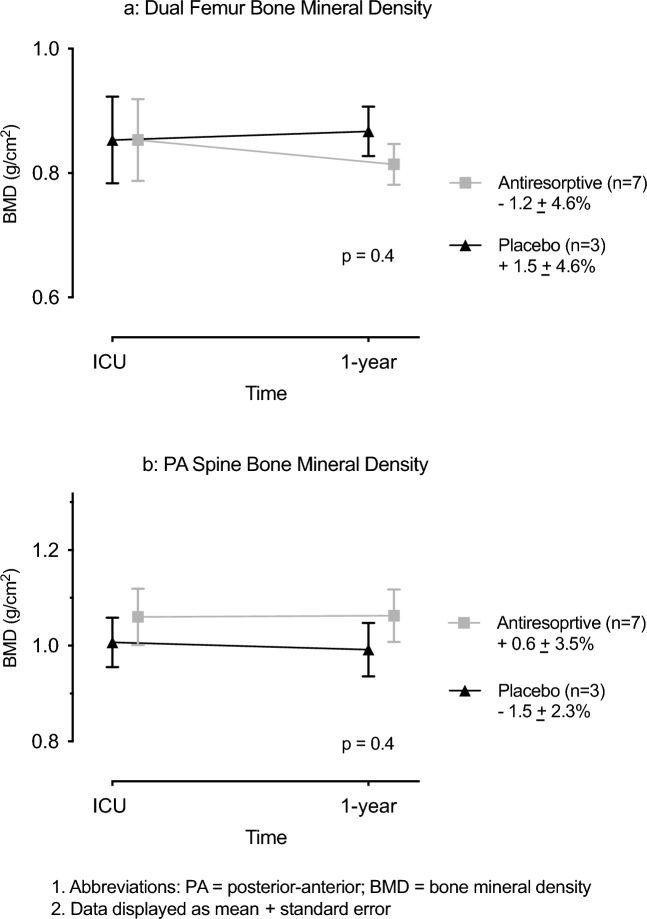
Change in bone mineral density over one year after critical illness.

Bone mineral density measurements at baseline and one-year were completed in 10 of 18 participants. Femoral neck BMD change was − 1.2 ± 4.6% in the antiresorptive group vs + 1.5 ± 4.6% in the placebo group, and spine BMD change was + 0.6 ± 3.5% in the antiresorptive group vs – 1.5 ± 2.3% in the placebo group (Fig. 4). Due to the small sample size statistical analysis was not performed.

Adverse events are provided in Table 3. Overall, no serious adverse events were reported. One adverse event was reported, asymptomatic hypocalcaemia in a participant in the placebo arm at the 6-month visit.

**Table 3:** Adverse events.

Outcome	Antiresorptive (n = 11)	Placebo (n = 7)
Serious adverse event		
Symptomatic hypocalcaemia	0 (0)	0 (0)
Osteonecrosis of the jaw	0 (0)	0 (0)
New infections	0 (0)	0 (0)
Adverse events		
Hypocalcaemia	0 (0)	1 (14.3)
Skin reaction	0 (0)	0 (0)
General*	0 (0)	0 (0)

*Abdominal pain, arthralgia, back pain, pain in extremity.

## Discussion

### Key findings

The administration of antiresorptive agents in critically ill women aged 50 years or above was associated with a significant short-term decrease in bone resorption markers, with no reported adverse events. Recruitment in this pilot trial, however, was slow, occurring at the rate of 1 participant every 2-months, an important feasibility issue for future studies.

### Relationship to previous studies

There is a clear rationale for systematically investigating the role of antiresorptive agents during critical illness. First, critical illness is associated with increased bone turnover, characterised by increased osteoclastic bone resorption, an increase in immature osteoblast number and activity, and reduced activity of mature osteoblasts, of the magnitude described in postmenopausal females or metabolic bone disease^9,10,19,20^. There is limited evidence describing the mechanism and cellular pathways associated with this relationship. The effect of cytokines on osteoclast activation factors that increase maturation and lifespan of osteoclasts, a direct effect of cytokines on osteoclast precursors has been described, and a positive association between critical illness related changes to the somatotrophic, thyrotrophic, and gonadotrophic axes, inflammatory cytokines and osteoclastic and osteoblastic activity has been described^19^. In addition, in-vitro experiments report an increased number and activity of osteoclast activation factors and mature osteoclasts, and reduction in bone angiogenesis factors and vascularity, when peripheral blood mononuclear cells (PBMCs) are exposed to sera of critically ill patients compared to healthy ill controls^10^.

Second, increased bone loss is associated with low BMD, fragility fractures, and associated morbidity and mortality in non-critically ill populations^1^. There is evidence of accelerated bone loss^7,11,12,21^, and an increased risk of fragility fractures^14^ in critically ill populations.

Third, available antiresorptive agents safely prevent bone loss, fracture, and subsequent mortality in non-critically ill populations^22–25^. Zoledronic acid is a parenterally available, annual dose, potent bisphosphonate. It is analogous in molecular structure to pyrophosphate and suppresses bone resorption by entering osteoclasts and binding and inhibiting the enzyme farnesyl pyrophosphate synthase, resulting in disruption of osteoclast attachment to bone surface. Zoledronic acid is a first-line agent in the prevention and treatment of osteoporosis^25,26^. Denosumab is a fully human monoclonal antibody that specifically binds receptor activator of nuclear factor-kappa B ligand (RANKL). RANKL is required for differentiation of osteoclast precursors to mature osteoclast, is abundantly expressed by osteoblasts, bone marrow stromal cells, and T and B-lymphocytes, and binds to RANK receptor on osteoclasts, stimulating activity. Denosumab inhibits the RANKL pathway differentiation of osteoclasts and bone resorption, reducing bone loss and preventing fracture^23,24,28^.

Finally, increased bone turnover is evident in the first days of critical illness^19^, justifying early intervention.

This trial adds safety, and feasibility data to the limited evidence relating to administration of antiresorptive agents—zoledronic acid and denosumab—in patients with critical illness.

The administration of these two antiresorptive medications in this study was associated with a pattern of suppression of bone resorption with no increase in bone formation, and adds to two previous small, single centre trials in similar populations. A randomised trial of administration of the short-acting bisphosphonate ibandronate in 20 chronically ventilated postmenopausal women, reported a significant reduction in serum CTX at day 6, with the effect disappearing by day 11^30^. In contrast, serum osteocalcin, a protein released by osteoclasts and important in bone mineralisation, increased similarly in both groups. A recent randomised controlled trial of early administration of denosumab in 14 ventilated women and men with severe intracerebral haemorrhage, reported an 80% decrease in serum CTX over 4-weeks with denosumab, compared to a 56% increase with placebo^20^. Denosumab was associated with a significant decrease in serum osteocalcin compared to placebo, with no difference in the bone formation marker P1NP.

The difference in parathyroid hormone, alkaline phosphatase, and phosphate observed between the groups over time is consistent with the activity of the antiresorptive agents. This could reflect a response to hypocalcaemia, or a more direct relationship with denosumab and the RANKL-osteoclast pathway^31^. Overall, these studies suggest antiresorptive agents administered in intensive care provide a significant but potentially short-lived effect on osteoclast activation and bone resorption, with a less clearly defined impact on osteoblast activation and bone formation.

There is no high-quality evidence reporting the relationship between administration of antiresorptive agents in critically ill populations and interval change in vertebral and hip bone density as an outcome. While bone turnover markers are useful to assess short-term efficacy of antiresorptive agents, their value is dependent on the association with BMD and fragility fracture rate^32^. The current evidence in critical illness is limited to a retrospective propensity matched analysis reporting an association between prior bisphosphonate use and reduced change in vertebral BMD assessed by serial CT scans in ICU patients^21^, and a prospective observational study describing the association between antiresorptive agents administered in the year after critical illness and reduced loss of spine BMD in women, and femur BMD in men^33^.

The use of change in BMD after critical illness represents a logistic challenge for intensive care research, as assessment should occur at a minimum 1-year interval, due to the short-term precision error of measurement by dual energy x-ray absorptiometry^33^. This trial is the first randomised controlled trial in a critically ill patient cohort to attempt to measure change in BMD over 1-year. The interruption of BMD activity and cessation due to the COVID-19 pandemic, resulted in insufficient sample size to enable meaningful statistical comparison.

This trial reported no adverse events related to trial medications. While the sample size is small, the absence of severe hypocalcaemia, infection, or acute renal failure is reassuring. Assessment of safety and adverse events should be examined in a larger trial.

### Strengths and limitations

This trial provides a clear, reproducible procedure for the safe administration of antiresorptive agents in a critically ill population and establishes a protocol for measuring both bone turnover markers and BMD. A major limitation was the inability to recruit the planned sample of 30 participants, limiting statistical analysis and the interpretation of these results. The COVID-19 pandemic made ongoing recruitment and follow-up in the Australian healthcare environment not feasible. In addition, the barriers, and facilitators to encourage inclusion, recruitment, and retention of women in clinical trials have been described in other settings^35,36^, and this issue has not been clearly examined in the critical care population.

## Conclusions

This trial reports the administration of antiresorptive agents to older critically ill women was associated with favourable short-term changes in bone resorption markers and no adverse events. This supports the need for larger trials in the critical illness setting to define the efficacy, duration and degree of effect, safety of antiresorptive agents, and effect on bone mineral density and fracture over at least 1-year of follow-up. Barriers to enrolment should be identified and addressed.

### Supplementary Information

Supplementary Information.

Supplementary Table 1.
