# Supplementary material for: A pilot feasibility randomised controlled trial of bone antiresorptive agents on bone turnover markers in critically ill women
Source: Sci Rep. 2024 Jan 24;14:2071. doi: 10.1038/s41598-024-52607-1 (PMC10810087; doi:10.1038/s41598-024-52607-1)
Supplement: Supplementary file 2 — Supplementary Table 1. [file 41598_2024_52607_MOESM2_ESM.docx]

**Supplement Table 1: Inclusion and Exclusion Criteria**

Inclusion criteria

1. Female
2. Age >50 years or post-menopausal (amenorrhea for greater than 6-months or serum FSH >40mIU/L) *or* age < 50 years with bilateral salpingo-oopherectomy
3. Intensive care unit length of stay > 24 hrs

Exclusion criteria

1. Active malignancy
2. Metabolic bone disease
3. Pregnancy
4. Current eGFR <30ml/min
5. Known contraindication to denosumab (previous reaction, osteonecrosis of the jaw, atypical femoral fracture)
6. Increased risk of osteonecrosis (poor dentition or oral hygiene, dental infection)
7. Hypoparathyroidism
8. Malabsorption sydnromes / extensive small bowel resection
9. Current treatment with anti-fracture agent (bisphosphonate, strontium, teriparatide, within previous 2 years or denosumab within previous 6 months)
10. Current indication for anti-fracture therapy (known BMD T-score < -2.5 and fragility fracture)
11. Death is imminent or expected in this hospital admission.
